# Natural and Synthetic Coumarins with Effects on Inflammation

**DOI:** 10.3390/molecules21101322

**Published:** 2016-10-02

**Authors:** Gilbert Kirsch, Ahmed Bakr Abdelwahab, Patrick Chaimbault

**Affiliations:** 1 SRSMC, UMR 7565, Groupe HeCRIN, ICPM, 1 boulevard Arago, 57070 Metz, France; ahmed.mahmoud@univ-lorraine.fr (A.B.A.); patrick.chaimbault@univ-lorraine.fr (P.C.); 2 Chemistry of Natural Compounds Department, National Research Centre, El-Behoos St. 33, 12622 Dokki-Cairo, Egypt

**Keywords:** coumarins, furocoumarins, pyranocoumarins, inflammation

## Abstract

In this review, we will present the different aspects of coumarins and derivatives, from natural origins or synthetically prepared, and their action on inflammation. Coumarins and also furo- and pyranocoumarins are found in many different plants. These compounds are very often investigated for antioxidant properties. Other biological properties are also possible and anti-inflammation activity is one of these. As coumarins are also available quite easily via synthesis, natural ones can be prepared this way but derivatives with special substituents are also feasible. A review on the same topic appeared in 2004 and our contribution will take into account everything published since then.

## 1. Introduction

Inflammation is a general and essential response to any aggression and may be shown by a localized response or by a more generalized one. Inflammation is thought to be at the start of different diseases including obesity, cancer and neurodegenerative illness. The biosynthetic cascade of arachidonic acid leading to the formation of prostaglandins (PG), leukotrienes (LT), hydroxyeicosatetraenoic acids (HETE) have been shown to be involved in the inflammation as well as other processes. Blocking the cascade is a possible way to fight inflammation. Inflammation is the process by which leucocytes and material derived from the serum are directed to the site of tissue injury. Coumarins are widespread natural chemicals [1]. Many of these compounds have a pharmacological use [2]. In this review we will investigate coumarins and derivatives with anti-inflammatory activity. We will present this study in terms of the chemical structure: simple coumarins, furo- and pyrano-coumarins.

In the first part, naturally occurring coumarins will be presented. The second part will be devoted to synthetic coumarins.

## 2. Naturally Occurring Coumarins

### 2.1. Simple Coumarins

**Figure 1:**
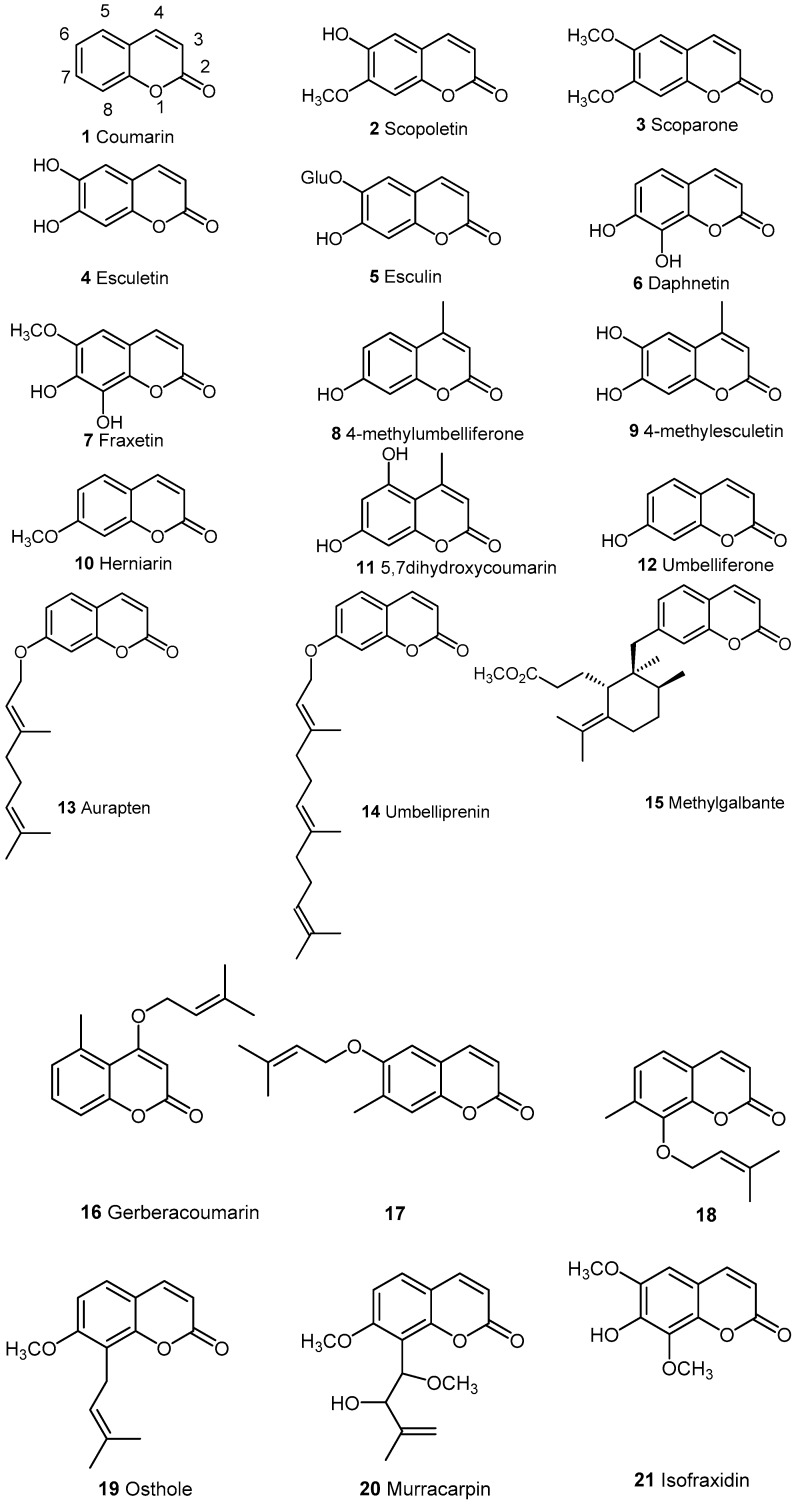
Natural coumarins.

Figure 1 shows the plant derived coumarins described in the literature with anti-inflammatory activities.

Coumarin **1** itself was used for the treatment of edema [3]. A general study on **2**, **3**, **5**, **6**, **7**, **8** has been run on TNBS (trinitrobenzenesulfonic acid) induced colitis [4]. The study showed that these coumarins are potential agents for treating the inflammation. The effect seems directly related to the antioxidant properties of the coumarins, in particular for daphnetin **6**, esculin **5** and scoparone **3**. Food containing coumarins could be a possible strategy to prevent Inflammation Bowel Diseases (IBD). Natural products like esculetin **4**, fraxetin **7**, daphnetin **6** and other related coumarin derivatives are recognized as inhibitors not only of the lipoxygenase and cycloxygenase enzymic systems, but also of the neutrophil-dependent superoxide anion generation [5].The study on the ethanolic extract of Artemisia capillaris Thunberg (Compositaea) used in traditional Chinese medicine for amelioration of skin inflammation showed inhibition activity on 5-lipooxygenase [6]. In particular, esculetin 4 had the property along with other compounds [6]. Experiments run on inflammation in animal models (xylene induced ear edema, carrageanan induced paw edema and carrageanan induced mouse pleuresy) [7] have shown that esculin **5**, a glycoside of esculetin, attenuated the phenomena. In-vitro pro-inflammatory cytokines levels of TNF-α and IL-6 were reduced by esculin. It was also found that esculin significantly inhibited LPS (lipopolysaccharide) induced activation of MAPK pathway in peritoneal macrophages. Esculin could be a promising agent for treating inflammatory diseases in humans. Another study on rat peritoneal leukocytes using 16 different coumarins [8] has pointed out the effect on LTB_4_ and TXB_2_ generated by calimycin (A23187). Fraxetin **7**, esculetin **4**, daphnetin **6**, 4-methylesculetin **9** were the most active on LTB_4_ and herniarin **10**, 4-methyl-5,7 dihydroxycoumarin **11** and daphnetin **6** on TXB_2_ with IC_50_ ranging from 1–75 µM. Daphnetin **6** appears often as an effective compound. The compound extracted from Daphnea odora was tested in collagen induced arthritis in rats [9]. The therapeutic effect was determined by the balance of Tregs and Th-17. The levels of these lymphocytes were evaluated by ELISA and those related receptors. by immunochemistry. Administration of daphnetin for 21 days highly alleviated the severity of arthritis by modulating the balance. In continuation looking at the power of daphnetin, some finding suggest that it might have a neuroprotective effect in stressed mice [10]. The role of daphnetin in microglial inflammatory response was explored. The study showed that all the pro-inflammatory mediators (IL-1B, TNFα induced by LPS) were strongly depressed in a dose dependent manner in BV_2_ microglia and it inhibited the LPS-induced iNOS and COX-2, even NO formation by microglia. In general daphnetin inhibits microglial activation and proinflammatory responses by modulating a series of intracellular pathways (IKK/κB,MAPKs and Pt.3K/Akt).

Umbelliferone **12** has not been found to have anti-inflammatory properties but some of its derivatives did. Among these, aurapten **13** and umbelliprenin **14** were the most studied. These compounds are found especially in the Ferula species. Aurapten **13** from Ferula szowitsiana showed cancer chemopreventive properties suggested by anti-inflammatory activity of the compound [11]. A mini-review on the biological activity of umbelliprenin from Artemisia species has appeared [12] linking the activity to iNOS inhibition. A paper [13] describing an extract from Ferula szowitsiana describes the activity of 6 terpenoïd coumarins isolated (methyl galbanate, galbanic acid, farnesiferol A, badrakemone, umbelliprenin and aurapten). In this case methylgalbanate **15** was the best inhibitor of nitric oxide production; it was better than umbelliprenin isolated from the same species.

Three naturally occurring O-prenyl coumarins; 4-isopentenyloxy-5-methyl-coumarin **16**, 6-isopentenyloxy, -7-methoxy-coumarin **17**, 8-isopentenyloxy-7-methoxy-coumarin **18**; from respectively Gerbera crocera and Gerbera serrata (Asteraceae), Haplophyllum pedicellatum (Rutaceae) and divers Artemisia species were prepared by synthesis [14]. They have been studied as dual anti-bacterial and anti-inflammatory compounds for controlling peritoneal diseases. U937-3xκB-LUC cell line was used to evaluate the ability to inhibit the activation of NF-κB signaling pathway induced by LPS. Compounds **17** and **18** were effective in the inhibition but not **16**. In fact, these two compounds also had anti-bacterial activity and were examples of dual action.

Osthole **19**, 8-prenyl-7-methoxy-coumarinapplied in clinical practice in Traditional Chinese Medicine is found in different plants (Angelica, Archangelica, Citrus, Clausena) and in high content in the mature fruit of Cnidium monnieri. The anti-inflammatory properties are described in a recent review ([15] and ref cited therein). Osthole has also been described as decreasing ocular inflammation [16].

Murracarpin **20**, structurally close to osthole and isolated from Murraya exotica showed strong anti-inflammatory and analgesic effect [17].

Ethylacetate extract of Canadian marple syrup contains many phenolics related compounds including coumarins. Fifteen pure compounds available were tested in LPS stimulated RAW264.7 murine macrophage and the decrease of nitric oxide and PGE_2_ measured. Unfortunately, the coumarinic isofraxidin **21** was not active [18]. All the biological results of simple coumarins are summarized in (Table 1).

### 2.2. Condensed Coumarins

The most condensed coumarins isolated from natural sources are furo- and pyranocoumarins. A review in 2006 [19] reports the biological activities of prenyloxy-furocoumarins and the in-vivo remarkable anti-inflammatory effect. We will review the publications appearing since then.

#### 2.2.1. Furocoumarins

**Figure 2:**
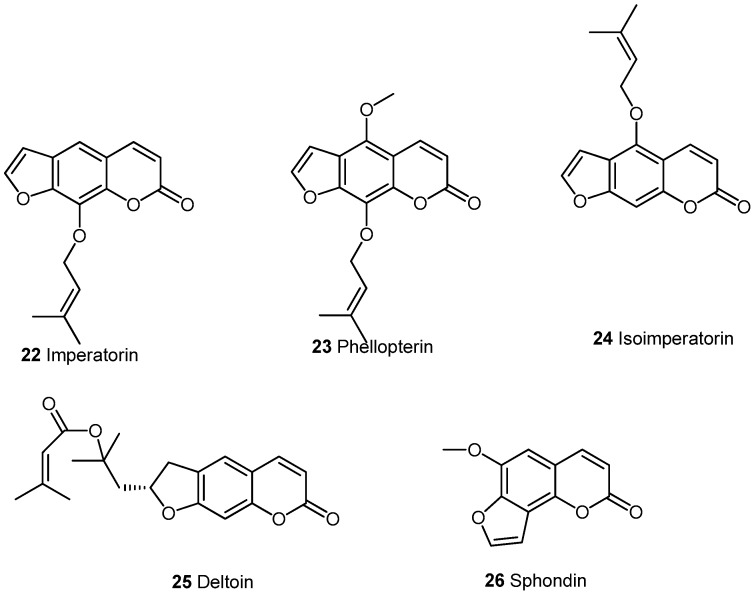
Naturally occurring furocoumarins.

Among furocoumarins (Figure 2), imperatorin 22 has been the most investigated. The substance is found in *Angelica dahurica, Glhenia littoralis, Cnidium monnieri and Peucedanum praeruptorum* as well as in other *Apiaceae* and *Umbellifereae* species. These plants are used in traditional Chinese and Korean Medicine. as antipyretic and analgesic. Most assays that investigated the behavior against inflammation were run on LPS activated RAW264.7 cells [20,21,22,23,24,25,26] and carrageanan induced mouse paw edema [21]. In general, inhibition of NOS as well as COX-2 were found to be the cause. Imperatorin has also proved to be efficient on allergic rhinitis [27], acute lung injury induced by LPS in mouse [28], on sebum production in human sebocytes in vitro [29] and it has had an anti-allergic effect on Th-2 mediated allergic asthma [30]. It has been stated that phellopterin 23 presents the same features as imperatorin concerning this activity [20]. Dimeric furocoumarins from the roots of *Angelica dahurica* had the same activity [31]. 

An isomer of imperatorin, isoimperatorin **24**, from the roots of *Angelica dahurica* [32] or *Cimicifugae* rhizome [33] was found to inhibit COX-dependent phases or TNF-α induced expression indicating its anti-inflammatory properties.

Various furocoumarins extracted from Chinese herbs were tested for iNOS 5 inhibition [34,35]. Among the different furocoumarins: imperatorin **22**, deltoin **25** and sphondin **26** gave the best results. As sphondin is also available in different *Heracleum* species it may provide a basis for the use of plant extracts against inflammation. For example, sphondin extracted from *Heracleum laciniatum* inhibits IL-1β induced PGE_2_ release in A549 cells [36]. This inhibition is mediated by suppressing of COX-2 expression and maybe at least in part through suppression of NF-κB activity. It is worth noting that the extract of fruits from *Heracleum crenatifolium* contains different furocoumarins and has anticonvulsivant activity in male albino mice [37]. A review on *Heracleum persicum* details the pharmacology of the furocoumarins in it [38].The biological results of furocoumarins are summarized in (Table 2).

#### 2.2.2. Pyranocoumarins

**Figure 3:**
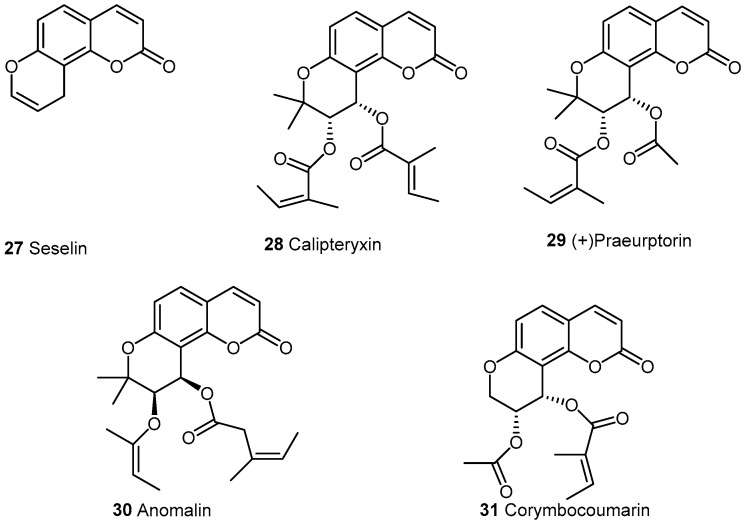
Naturally occurring pyranocoumarins.

Pyranocoumarins (Figure 3) have less been tested for this activity. The first compound of this class extracted was seselin **27** from *Sigmathantus trifoliatus* able to inhibit inflammatory hyperalgesia and having also antinociceptive effect [39]. Obtained from *Seseli resinosum*, calipteryxin **28** and a dihydroseselin derivative were associated in the inhibition of inflammatory enzymes (iNOS and COX-2) and cytokines (TNF-α and IL-6) via NF-κB, MAPK and Akt pathways [40]. (+)-Praeruptorin **29** from the roots of *Peucedanum praerupotorum* reduced NO production and release of IL-1β, IL-6 and TNF-α induced by LPS-stimulated macrophage [41,42]. Other derivatives anomalin **30** and corymbocoumarin **31** were obtained respectively from *Saposhnikovia divaricata* and *Seseli gummniferum* have been used in LPS-stimulated RAW264.7 macrophages [43]. Anomalin dose-dependently inhibited iNOS, COX-2 mRNA and protein expression. Concerning corymbocoumarin, it exerts its effect by suppressing NF-κB activation and inducing HO-1 expression [44]. The biological results of this kind of coumarins are summarized below in (Table 3).

## 3. Synthetic Coumarins and Pyranocoumarins

**Figure 4:**
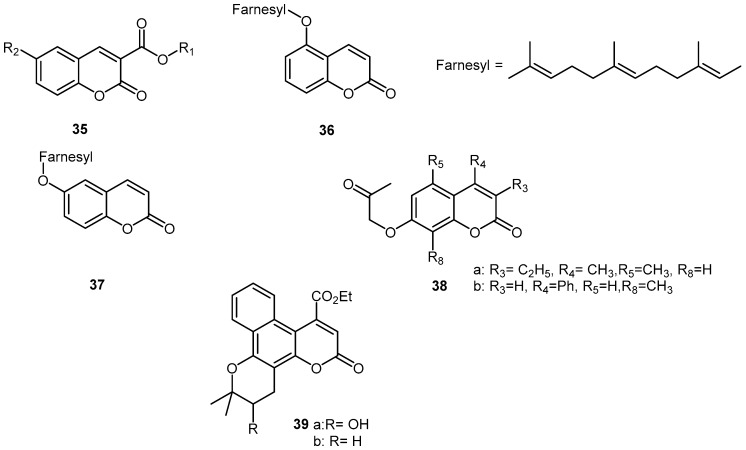
Synthetic coumarins and derivatives.

**Figure 5:**
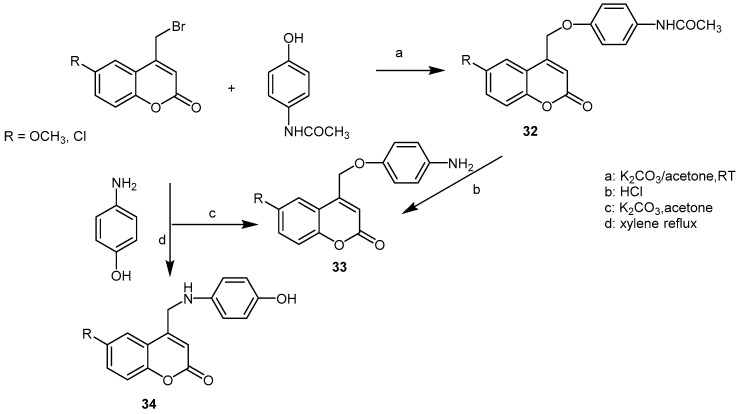
Synthesis of paracetamol modified coumarins.

As functionalized coumarins can be easily prepared through synthesis, different compounds not naturally found have been prepared and tested (Figure 4). Starting from 6-chloro and 6-methoxy 4-bromomethyl-coumarin, paracetamol ethers **32**, **33**, **34** have been prepared (Figure 5).

Screening the activity using carrageenan induced edema in rats gave the best results for compounds **32** where R = Cl and OCH_3_ was in position 6 [45]. A series of esters prepared from coumarin-3-carboxylic acids **35** obtained from the corresponding salicaldehydes, were tested in LPS-induced lung inflammation and elastase induced lung injury [46]. The data obtained showed that all coumarins had anti-inflammatory properties and that anti-elastase activity is essential to reducing lung inflammation in-vivo. For looking at 15-LPO inhibitors, a series of *O*-prenylated coumarins were synthesized (isopentyloxy, farnesyloxy, geranyloxy in 4, 5, 6, 7, and 8 position) [47]. 5-Farnesyloxycoumarin **36** showed the most potent activity against soybean lipoxygenase and 6-farnesyloxycoumarin **37** against human lipoxygenase. A great number of 7(2-oxoalkoxy)coumarins **38** have been prepared by condensation of hydroxycoumarins with α-chloroacetone [48] and the effects on expression of iNOS and COX-2 measured in comparison with 7-hydroxycoumarin. Result was that the substitution increased the activity compared to the unsubstituted one. Most active compounds were **38a** and **38b**. As it has been demonstrated, gold nanoparticles can reduce the expression of pro-inflammatory molecules [49], the idea to conjugate scopoletin **2** to gold nanoparticules emerged [50]. Scopoletin from *Artemisia roxburghiana* was conjugated in situ to gold nanoparticules prepared by reduction of tetrachloroauric acid. The NO inhibitory activity of scopoletin was unaffected by the conjugation (NOS was induced by LPS on J.774.2cell lines). However, the conjugation increased the prevention of oxidative burst of ROS in the whole blood phagocytes and isolated neutrophiles.

A methodology to prepare coumarins from ortho-benzoquinones has been used for the synthesis of benzopyranocoumarins **39** [51,52,53] analogs to benzo[1]khellactone. All tested compounds [52], highly inhibited soybean lipoxygenase, competing with DMSO for OH^•^ and some induced protection against carrageenan induced rat paw edema. Another series [53] showed similar results for competition with DMSO for OH^•^, for O_2_^−^ scavenging and lipoxygenase inhibition. The biological results of synthetic coumarins are summarized in (Table 4).

## 4. Conclusions

Coumarins and derivatives, even those that have been known for a long time, are still a source of interesting biological active compounds. As the synthesis of these compounds is quite easy, access to a greater amount of derivatives is possible. Since the compounds are sometimes present in edible foods, these may serve as anti-inflammatory supply and as fiber containing plants are considered prebiotics this may help even more to fight inflammation.

## Figures and Tables

**Table 1:** Some anti-inflammatory activities of Simple coumarins.

Cpd	CPE% ^1^	IL-1β	IL-6	5-LOX	NF-κB	NO	TNF-α	Ref.
**4**				IC_50_: 6.6 ^2^				[5]
**5**			<200 ng/mL (20 mg/kg) ^3,^**				<400 ng/mL (20 mg/kg) ^3,^**	[7]
**6**		<3000 pg/mL (160 μg/mL, 24 h) ^4,^*; <1000 pg/mL (160 μg/mL) ^5,^**			Activity < 1 (160 μM) ^4,^*	<20 μM (160 μM) ^4,^**	<500 pg/mL (160 μg/mL, 24 h) ^4,^*; <400 pg/mL (160 μg/mL) ^5,^**	[10]
**14**	39% (0.01 mmol/kg)			IC_50_: 72.5 nm				[12]
**15**						<10 μM (10 μM) ^6,^*		[13]
**16**					>80% (50 μg/mL) ^1^			[14]
**17**					<10% (50 μg/mL) ^1^			[14]
**18**					<5% (50 μg/mL) ^7^			[14]
**21**					0% ^6^	0% ^6^	0% ^6^	[18]

^1^ Carageenin paw edema in rat; ^2^ Arachidonic acid induced ear edema in mice; ^3^ LPS-stimulated mouse peritoneal macrophage; ^4^ LPS-stimulated BV2 microglia; ^5^ Aβ-stimulated BV2 microglia; ^6^ LPS-stimulated macrophage RAW264.7; ^7^ LPS-stimulated U937-3xxKB-LUC human monocytic cell line; kg is referring to the mice weight; % is referring to percentage of inhibition. * *p* < 0.05 versus negative control. ** *p* < 0.01 versus negative control.)

**Table 2:** Some anti-inflammatory activities of furocoumarins.

Cpd	COX-2	IL-1β	IL-6	IL-10	NF-κB	NO	iNO	PGE_2_	TNF-α	Ref.
**22**	<40% (10 μg/mL) ^3,^***	<20 pg/mL (10 μg/mL) ^1,^*; <300 pg/mL (30 mg/kg) ^2,^*	<600 pg/mL (30 mg/kg) ^2,^*	<600 pg/mL (15 mg/kg) ^2,^**		<30 μM (10 μg/mL) ^3,^***; <6 μM (10 mg/kg) ^4^; IC_50_: 52.3 ± 5.4 μM ^3,^***	<40% (10 μg/mL) ^3,^***; 83.3% (20 μg/mL) ^3^	<400 pg/mL (10 μg/mL) ^3,^***; <80 pg/mL (10 mg/kg) ^4,^***	<40 μM (10 ng/mL) ^3,^***; <300 ng/mL (10 mg/kg) ^4^; <1500 pg/mL (30 mg/kg) ^2,^**	[21,22,27,28,34]
**23**						IC_50_: 38.3 ± 1.7 μM ^3,^***				[22]
**24**	IC_50_: 10.7 μM ^5^					IC_50_>20 μg/mL ^3^, 28.1% ± 39.7% (20 μg/mL) ^3^				[32,35]
**25**						<20% (20 μg/mL) ^3,^****	92.4% (20 μg/mL) ^3^			[34]
**26**					<60% at 50 μM ^6,^*	IC_50_: 9.8 μg/mL ^3^; 85.3% ± 8.7% (20 μg/mL) ^3^	<5 μM (20 μg/mL) ^3^**	<10 ng/mL (50 μM) ^6,^*		[35,36]

^1^ PMACI induced of HMC-1 cells; ^2^ LPS-induced of BALF (broncoalveolar lavage fluid); ^3^ LPS-stimulated macrophage RAW264.7'; ^4^ Carageenin paw edema in rat; ^5^ In bone marrow-derived mast cell (BMMC); ^6^ IL-1β induced of A549 cells; kg is referring to the mice weight; % is referring to percentage of inhibition; * *p* < 0.05 versus negative control; ** *p* < 0.01 versus negative control; *** *p* < 0.001 versus negative control; **** *p* < 0.005 versus negative control.

**Table 3:** Some anti-inflammatory activities of pyranocoumarins.

Cpd	COX-2	IL-1β	IL-6	NF-κB	NO	iNO	PGE_2_	TNF-α	Ref.
**28**	<20% (30 μM) ^1,^***	<40% (30 μM) ^1,^***			<20 μM (30 μM) ^1,^***; <40 μM (30 μM) ^2,^**	<20% (30 μM) ^1,^***		<40% (30 μM) ^1,^***	[40]
**29**		<100 pg/mL (25 μg/mL) ^1,^**	<100 pg/mL (100 μM) ^1,^**		<15 μM (25 μg/mL) ^1,^**			<10000 pg/mL (25 μg/mL) ^1,^*	[41,42]
**30**	<60% (50 μM) ^1,^***			<4000 RFU (50 μM) for NF-κB-dependent alkaline phosphate (SEAP ) ^1,^***		<60% (50 μM) ^1,^***		<60% (50 μM) ^1,^***	[43]
**31**				<8000 RFU (60 μg/mL) ^1,^**	<20 μM (60 μM) ^1,^***		<500 pg/mL (60 μM) ^1,^***		[44]

^1^ LPS-stimulated macrophage RAW264.7; ^2^ SNP induced macrophage RAW264.7; % is referring to percentage of inhibition; * *p* < 0.05 versus negative control; ** *p* < 0.01 versus negative control; *** *p* < 0.001 versus negative control.

**Table 4:** Some anti-inflammatory activities of synthetic coumarins.

Cpd	COX-2	CPE% ^3^	IL-6	LOX	NO	iNO	TNF-α	Ref.
**32**, R = 6-Cl		69 (3 h); 60 (6 h) *						[45]
**35**, R1 = 5-chloropyridin-3-yl; R2 = CH_3_COOCH_2_			<600 pg/mL (1 μM/kg) ^1,^*				<1200 pg/mL (1 μM/kg) ^1,^*	[46]
**38a**	−8 ± 17.4 (10 μM) ^2^; 37 ± 15.2 (100 μM) ^2^		IC50: 10 μM ^2^; 33 ± 4.6 (10 μM) ^2^; 89 ± 0.7 (100 μM) ^2^		IC30: 30 μM ^2^; 49 ± 2 (10 μM) ^2^; 90 ± 1.6 (100 μM) ^2^	58 ± 5.5 (10 μM) ^2^; 99 ± 0.1 (100 μM) ^2^		[48]
**38b**	29 ± 7.8 (10 μM) ^2^ ; 57 ± 1.6 (100 μM) ^2^		IC50: 24 μM ^2^; 47 ± 4.5 (10 μM) ^2^; 90 ± 0.4 (100 μM) ^2^		IC50: 21 μM ^2^; 27 ± 3.6 (10 μM) ^2^; 89 ± 0.4 (100 μM) ^2^	47 ± 7.2 (10 μM) ^2^; 97 ± 0.6 (100 μM) ^2^		[48]
**39a**, R = OH		48.7 (0.1 mmol/kg, 3.5 h)		89.8% (1 mM)				[52]
**39b**, R = H		54 (0.1 mmol/kg, 3.5 h)		No (1 mM)				[52]

^1^ LPS induced alveolar macrophage; ^2^ J774 macrophage stimulated by LPS; ^3^ Carageenin paw edema in rat; kg is referring to the mice weight; % is referring to percentage of inhibition. * *p* < 0.05 versus negative control.
